# Supplementary material for: Energy-level quantization and single-photon control of phase slips in YBa2Cu3O7–x nanowires
Source: Nat Commun. 2020 Feb 7;11:763. doi: 10.1038/s41467-020-14548-x (PMC7005758; doi:10.1038/s41467-020-14548-x)
Supplement: Supplementary file 1 — Supplementary Information [file 41467_2020_14548_MOESM1_ESM.pdf]

Supplementary Information

Energy-level quantization and single-photon control of phase slips in $YBa_2Cu_3O_{7-x}$ nanowires

M. Lyatti et al.

Supplementary figures

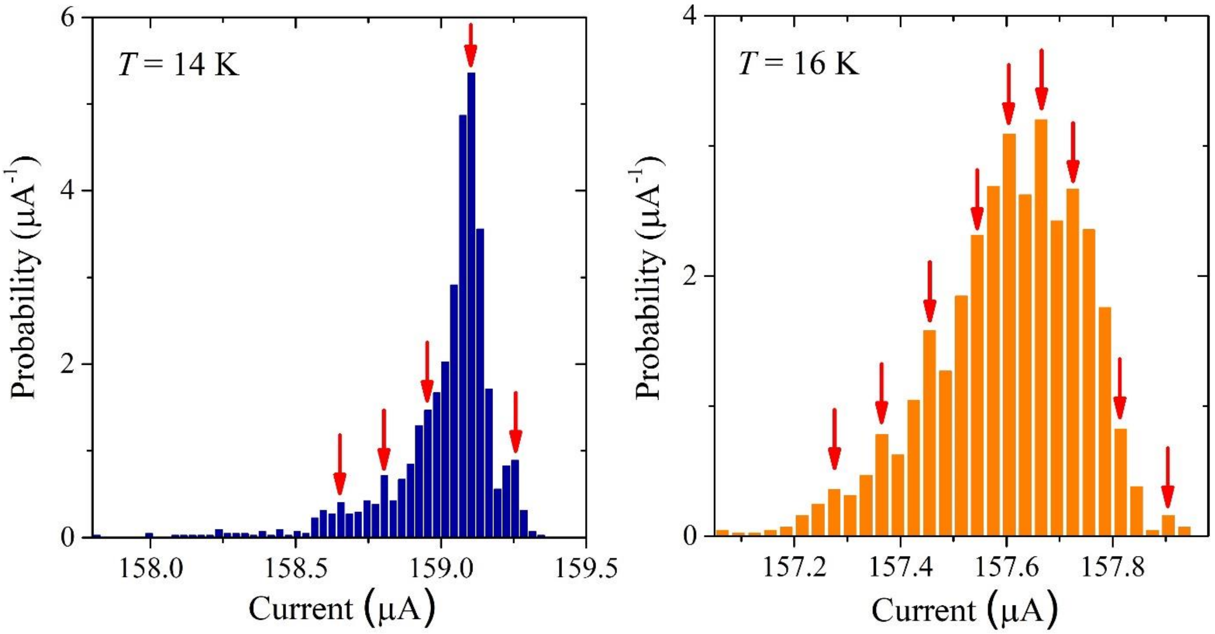

Supplementary Figure 1| Switching-current distributions for a 55-nm-wide YBCO nanowire measured under equilibrium conditions at temperatures of 14 and 16 K. Red arrows indicate the bias current value at which the height of the energy level coincides with the barrier height. Source data are provided as a Source Data file.

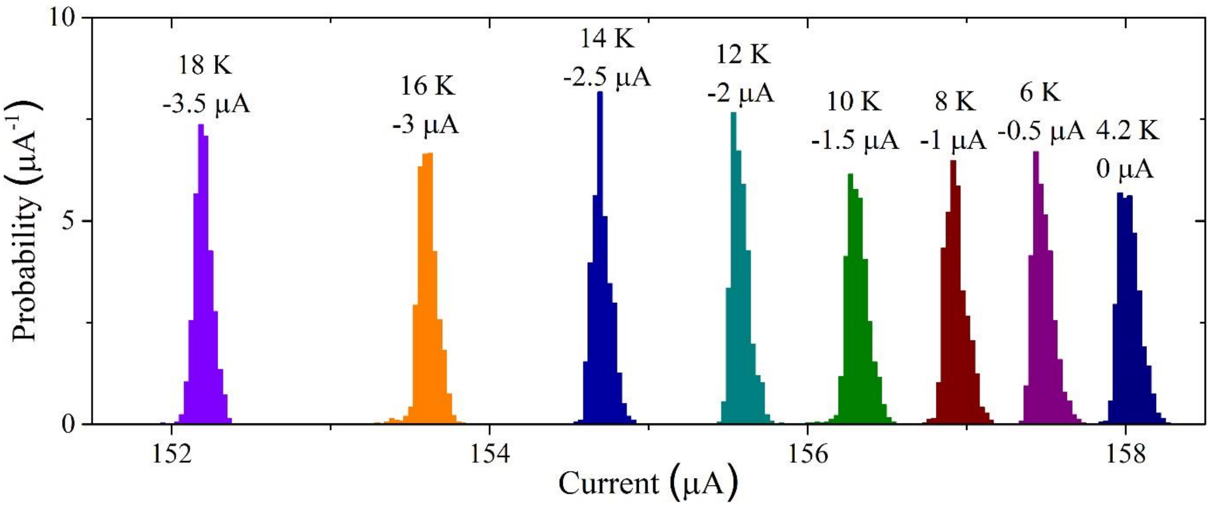

Supplementary Figure 2| Retrapping-current distributions for a 55-nm-wide YBCO nanowire measured under equilibrium conditions. The numbers above the peaks are the nanowire temperature and the shift along the current axis. Source data are provided as a Source Data file.

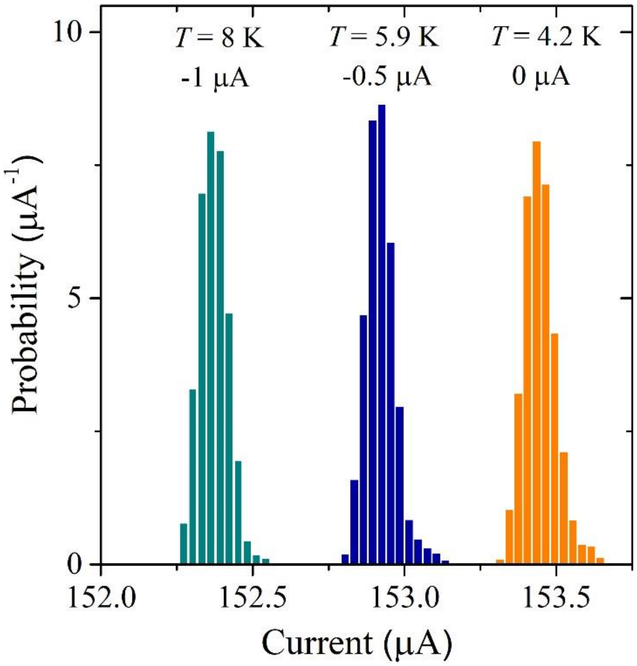

Supplementary Figure 3| Noise-affected retrapping current distributions for a 55-nm-wide YBCO nanowire. The numbers above the peaks are the nanowire temperature and the shift along the current axis. Source data are provided as a Source Data file.

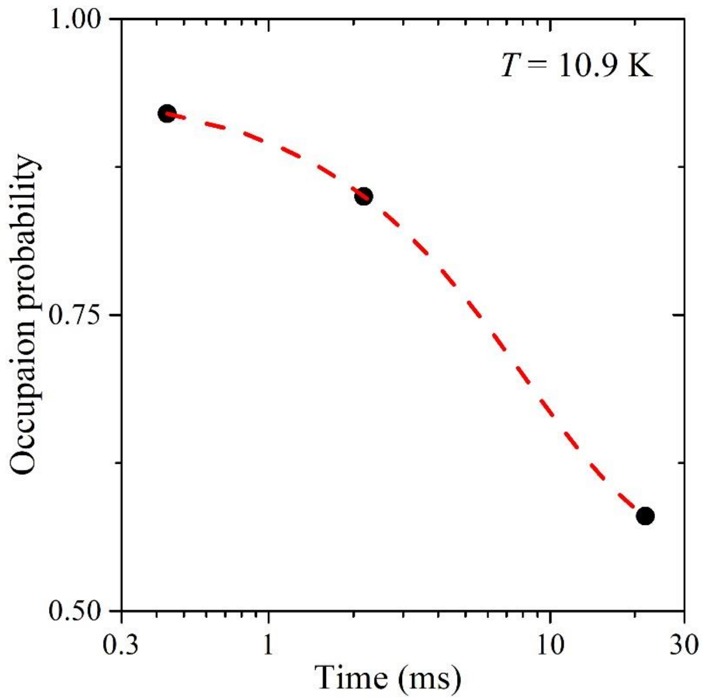

Supplementary Figure 4| Time dependence of the occupation probability of the excited state for a 55-nm-wide YBCO nanowire at 10.9 K (black dots). The dashed line is an exponential decay fit to the experimental data. Source data are provided as a Source Data file.

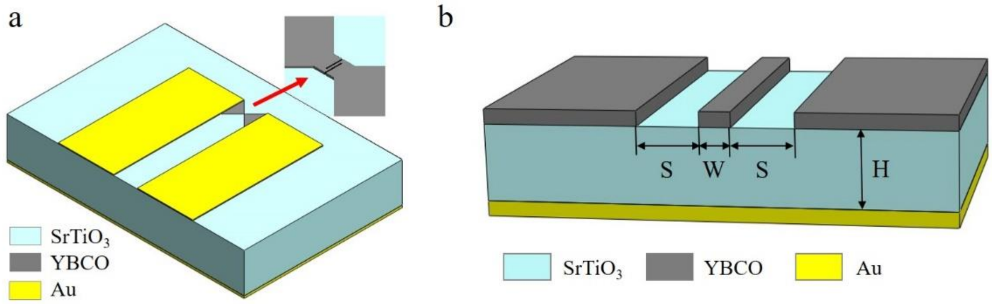

Supplementary Figure 5| a, Sketch of the whole nanowire device. **b**, Nanowire layout for capacitance calculations.

Supplementary Notes

Supplementary Note 1: Electrodynamics of YBCO phase-slip nanowires

In order to explain the measured switching-current statistics we analyze the electrodynamics of our YBCO nanowires in terms of the resistively and capacitively shunted junction (RCSJ) model, originally developed to describe 1D superconducting weak links featuring the Josephson effect. Here we show that the approximations of the RCSJ model are also valid for our YBCO phase-slip nanowires (PSN) and only minor modifications to the model are required to account for the difference between a PSN and a JJ. In correspondence with the devices studied in our work we consider a wide ($W >> \xi$) nanowire with significantly reduced critical current density J_c ($J_c << J_{GL}$) due to order parameter fluctuations. Here, W is the nanowire width, ξ is the coherence length, and J_{GL} is the Ginzburg-Landau depairing current density. The resistive state of the nanowire occurs due to a phase slippage by a kinematic vortices motion. We further consider that the capacitance is uniformly distributed along the nanowire.

Within the framework of the RCSJ model [1-3], the total current through a JJ is considered as a sum of a supercurrent I_s, a normal current I_n, a displacement current I_d, and current fluctuations $\delta I(t)$ as

$$I + \delta I(t) = I_s + I_n + I_d \quad (1).$$

The junctions resistance, R, and capacitance, C, are assumed as voltage-, frequency- and temperature-independent, such that $I_n = V/R$ and $I_d = C\mathrm{d}V/\mathrm{d}t$, where V is the voltage across the junction. The dependence of the superconducting current on the phase difference between superconducting electrodes φ is given by $I_s = I_c\sin(\varphi)$, where I_c is the fluctuation-free critical current. Using the voltage-phase Josephson relationship $h(\mathrm{d}\varphi/\mathrm{d}t) = 2eV$ Supplementary Eq. (1) can be rewritten as

$$I + \delta I(t) = I_c\sin(\varphi) + (h/2e)(1/R)(\mathrm{d}\varphi/\mathrm{d}t) + (h/2e)C(\mathrm{d}^2\varphi/\mathrm{d}t^2) \quad (2),$$

where $h = 2\pi\hbar$ is Planck's constant and e is the electron charge.

First, we consider the superconducting state of the YBCO PSN and show that it can be described by Supplementary Eq. (2). As long as the frequencies of all characteristic processes are small compared to $2\Delta/h$, where Δ is the superconducting energy gap, and there are no vortices in the nanowire, the current through the nanowire within the framework of the "two-fluid" model [4] can be approximated by a sum of the superconducting and normal components which is similar to Supplementary Eq. (1) of the RCSJ model. Note that in the superconducting state the voltage-phase Josephson relationship $h(d\varphi/dt) = 2eV$ holds for our YBCO PSN [1].

Based on the geometry of our 2-μm-long nanowire on an $SrTiO_3$ substrate we expect a resonance at a frequency of 6.1 THz, which is close to Δ/e = 6 THz. At Josephson plasma frequencies $\omega_p/2\pi \ll \Delta/e$, which are of practical interest in here, the nanowire can be considered as a lumped element where the normal and displacement currents can be expressed as $I_n = V/R_{nw} = (h/2e)(1/R_{nw})(d\varphi/dt)$ and $I_d = C_{nw}dV/dt = (h/2e)C_{nw}(d^2\varphi/dt^2)$, respectively. Here C_{nw} is the nanowire capacitance and R_{nw} is the nanowire resistance. Hence, the expressions for the normal and displacement currents resemble those in Supplementary Eq. (2). Note that in the superconducting state, the major contribution to the losses is due to unpaired quasiparticles. Assuming that the quasiparticles are in a thermal equilibrium with a bath temperature, we thus set $R_{nw} = R_{qp} = R_n e^{\Delta/kT}$, where R_n is the normal-state nanowire resistance, k is the Boltzmann constant, and T is the temperature.

The current-phase relationship (CPR) of a "conventional" superconducting nanowire, *i.e.* where fluctuations of the order parameter do not play any significant role, has a characteristic "sawtooth" shape [4] in striking difference from the sinusoidal CPR employed in the RCSJ model. However, if fluctuations of the order parameter cannot be neglected, the nanowire CPR will differ from such "sawtooth" shape [5,6]. Matveev et al. have predicted that the shape of the CPR transforms from the

“sawtooth” to the sinusoidal case as the order parameter fluctuations become stronger [5]. This transition has experimentally been observed by Arutyunov et al. [7]. Recently, Khlebnikov showed that the activation barrier in phase-slip nanowires with order parameter fluctuations obeys $\varepsilon = 2^{1/2}(1-i_b)^{1/2}+O[(1-i_b)^{3/2}]$, where $i_b = I/I_c$, and hence, at $I \rightarrow I_c$ the activation barrier scales as $(1-i_b)^{3/2}$ [6]. The $(1-i_b)^{3/2}$-scaling of the activation barrier is characteristic for Josephson junctions with sinusoidal CPR [4]. Hence, even for PSNs, where the complete transformation of the CPR from “sawtooth” to sinusoidal does not generally occur, a sinusoidal CPR is expected at bias currents close to the critical current. In this regard Arpaia et al. showed that the CPR of 50-nm-wide and 50-nm-thick YBCO nanowires is sine-like [8], meaning that it undergoes at least a partial transformation. Therefore, we assume that the CPR of our 5-nm-thick nanowires, which have 5-10 times smaller cross section than those in Ref. [8], are also of sinusoidal shape, at least at currents close to the switching current $I_s(I \rightarrow I_c) = I_c\sin(\varphi)$, where our experiments were performed. From the above discussion, we conclude that the electrodynamics of the YBCO PSN in the superconducting state can be described by an equation similar to that of Supplementary Eq. (2) of the RCSJ model. Hence, we apply the formalism of the RCSJ model for calculating the zero-bias plasma frequency $\omega_{p0} = (2eI_c/\hbar C_{nw})^{1/2}$, the quality factor in the superconducting state $Q_s = \omega_{p0}R_{qp}C_{nw}$, and the height of the potential barrier $\Delta U(I) = (hI_c/2\pi e)[(1-(I/I_c)^2)^{1/2}-(I/I_c)\arccos(I/I_c)]$ for our YBCO PSNs in the superconducting state.

The resistive state of a PSN that is shunted by a large capacitor, on the other hand, resembles that of an underdamped JJ [9]. The escape from one local potential minimum to another lower-lying potential minimum is accompanied by the 2π phase slip. This charges the capacitor and stimulates further phase evolution in accordance with the voltage-phase Josephson relationship

$d\varphi/dt = (2e/h)V$. Thus, a single escape event switches the capacitor-shunted nanowire into the resistive state, similar to the "running" state of an underdamped JJ.

However, there are important distinctions between "running" states of JJs and PSNs. Firstly, the normal-state resistance R of the JJ controls the discharge rate of the corresponding capacitor. For a PSN on the other hand, the capacitor's discharge rate is controlled by the resistance of the phase-slip line, which can be calculated as $R_{ps} = dV/dI$ [4]. Secondly, if the nanowire length $L >> \Lambda_Q$, it is a good approximation to consider that the current induced by the phase-slip oscillations flows only in the resistive part of the nanowire with length of $2\Lambda_Q$, due to the impedance mismatch between the resistive and superconducting part of the nanowire at the frequency of the phase-slip oscillations. Here, Λ_Q is the charge imbalance distance. Therefore, only the part of the nanowire capacitance with a value of $(2\Lambda_Q/L)C_{nw}$ will be charged and the quality factor of the nanowire in the resistive state is given as $Q_n = (2\Lambda_Q/L)\omega_{ps}R_{ps}C_{nw}$, where $\omega_{ps} = (2e/h)V_s$ is the frequency of the phase-slip oscillations, and V_s is the voltage switching amplitude. Supplementary table 1 summarizes the parameters of the extended RCSJ model that we use for analyzing our experimental data.

	Superconducting state	Resistive state
Characteristic frequency	Josephson plasma frequency<br>$\omega_p = (2eI_c/\hbar C_{nw})^{1/2}$	Frequency of phase-slip oscillations<br>$\omega_{ps} = (2e/h)V_s$
Losses	Quasiparticle resistance<br>$R_{qp} = R_n e^{\Delta/kT}$	Resistance of the phase-slip line<br>$R_{ps} = dV/dI$
Quality factor	$Q_s = (2eI_cC_{nw}/\hbar)^{1/2}R_{qp}$	$Q_n = (2e/h)(2\Lambda_Q/L)V_sR_{ps}C_{nw}$
Potential barrier height	$\Delta U(I) = (hI_c/2\pi e)[(1-(I/I_c)^2)^{1/2}-(I/I_c)\arccos(I/I_c)]$	-

Supplementary table 1| Parameters of the extended RCSJ model describing the electrodynamics of the YBCO phase-slip nanowire.

Supplementary references

1 Likharev, K. K. Superconducting Weak Links. *Rev. Mod. Phys.* **51**, 101-159 (1979).

2 Mccumber, D. E. Effect of Ac Impedance on Dc Voltage-Current Characteristics of Superconductor Weak-Link Junctions. *J. Appl. Phys.* **39**, 3113-3118 (1968).

3 Stewart, W. C. Current-Voltage Characteristics of Josephson Junctions. *Appl. Phys. Lett.* **12**, 277 (1968).

4 Tinkham, M. *Introduction to superconductivity*. (McGraw-Hill, Inc., 1996).

5 Matveev, K. A., Larkin, A. I. & Glazman, L. I. Persistent current in superconducting nanorings. *Phys. Rev. Lett.* **89**, 096802 (2002).

6 Khlebnikov, S. Decay of near-critical currents in superconducting nanowires. *Phys. Rev. B* **94**, 064517 (2016).

7 Arutyunov, K. Y., Hongisto, T. T., Lehtinen, J. S., Leino, L. I. & Vasiliev, A. L. Quantum phase slip phenomenon in ultra-narrow superconducting nanorings. *Sci. Rep-Uk* **2**, 293 (2012).

8 Arpaia, R. *et al.* Ultra low noise $YBa_2Cu_3O_{7-\delta}$ nano superconducting quantum interference devices implementing nanowires. *Appl. Phys. Lett.* **104**, 072603 (2014).

9 Belkin, A., Belkin, M., Vakaryuk, V., Khlebnikov, S. & Bezryadin, A. Formation of Quantum Phase Slip Pairs in Superconducting Nanowires. *Phys. Rev. X* **5**, 021023 (2015).
